# Effects of ambient temperature on ambulance emergency call-outs in the subtropical city of Shenzhen, China

**DOI:** 10.1371/journal.pone.0207187

**Published:** 2018-11-12

**Authors:** Zhi-Ying Zhan, Yi-Min Yu, Jun Qian, Yun-Feng Song, Ping-Yan Chen, Chun-Quan Ou

**Affiliations:** 1 State Key Laboratory of Organ Failure Research, Department of Biostatistics, Guangdong Provincial Key Laboratory of Tropical Disease Research, School of Public Health, Southern Medical University, Guangzhou, China; 2 Shenzhen Center for Prehospital Care, Shenzhen, China; 3 The People's Hospital of Longhua, Shenzhen, China; 4 Department of Mathematics and Physics, School of Biomedical Engineering, Southern Medical University, Guangzhou, China; 5 Intensive Care Unit, Guangdong No.2 Provincial People’s Hospital, Guangzhou, China; Ball State University, UNITED STATES

## Abstract

The associations between meteorological factors and mortality have been well documented worldwide, but limited evidence is available for the non-fatal health impacts of ambient temperature, particularly there are few population-based investigations on the impacts of emergency ambulance dispatches in Asia. In this study, based on 809,906 ambulance emergency call-outs (AECOs) for the total population from 2010–2016 in the subtropical city of Shenzhen, China, a Poisson regression combined with a distributed lag nonlinear model was used to simultaneously assess the nonlinear and lag effects of daily mean temperature on AECOs. Stratified analyses by age and sex were performed to identify vulnerable subpopulations. A U-shaped relationship was found between temperature and AECOs. Cold effects were delayed and persisted for 3–4 weeks, with a cumulative relative risk (RR) and 95% confidence interval (CI) of 1.23 (1.10–1.38) and 1.25 (1.16–1.35) over lag 0–28 when comparing the 1st and 5th percentile of the temperature distribution to the optimal (i.e. minimum AECOs) temperature, respectively. Hot effects were immediate and diminished quickly in 5 days, with an increase of 19% (RR = 1.19, 95%CI: 1.14–1.23) and 21% (RR = 1.21, 95%CI: 1.16–1.26) in AECOs over lag 0–5 when comparing the 95th and 99th percentile of temperature to the optimal temperature. Children and the elderly were more vulnerable to cold effects. The youth and middle-aged people suffered more from high temperature. The effects of temperature were similar between males and females. In summary, significant increases were observed in the frequency of AECOs during cold and hot days, and the weather-associated increases in AECOs are different among age groups. This information has valuable implications in ambulance demand prediction and service provision planning.

## Introduction

The associations between meteorological factors and mortality have been well documented worldwide [1–6]. Fatal impacts at the top of the health impact pyramid only represent the extreme impacts. In recent years, non-fatal impacts of ambient temperatures are of increasing concern. However, compared with the literature on the effects on mortality, the literature on the effects on medical emergency needs has been limited because, unlike mortality registration, an integrated data platform of medical emergency services common to all hospitals is absent in most cities, causing difficulties in obtaining medical emergency data for the total population.

The literature on the effects of medical emergency have been mainly based on the data of emergency department (ED) visits from one or several hospitals in a city, showing the effects of ambient temperature on ED visits for all causes and some specific causes (e.g., cardiovascular diseases, asthma, accidental casualties, middle ear inflammation) in Croatia, South Korea and Italy [7–9]. Zhao et al [10] also showed temperature-associated ED visits in 12 Chinese cities based on one-hospital data in each city. Two studies in Taiwan [11] and Shanghai [12], have utilized health data from their respective health insurance systems to investigate the temperature-associated ED visits.

Neither the hospital- nor insurance system-based studies have covered all ED visits for the total population. Additionally, the coverage proportion may be unstable over time, probably leading to selection bias and inaccurate estimates of temperature effects. Thus, a population-based investigation would provide a more comprehensive insight into ambient temperature-associated emergency needs.

Ambulance services in urban areas constitute the first line of demands and utilizations of Emergency Medical Services (EMS). The EMS system provides information on ambulance dispatch data, allowing us to examine the effects of temperature on medical emergency needs. A few studies have shown an increase in the number of ambulance emergency call-outs (AECOs) during heat waves in Birmingham, United Kingdom (UK) [13] and New South Wales [14]. A high temperature-related ambulance call increase was also observed in Australia [15] and Hong Kong [16].

Less is known about the impacts of low temperature on ambulance calls. The increased number of calls associated with cold temperature was observed in London, a temperate city [17], however, Yang et al. [18] did not observe significant effects of relatively low temperature on daily emergency ambulance dispatches for renal colic in the subtropical city of Guangzhou, China. Thus, we need to further investigate the exposure–response association between temperature and all AECOs and, in particular, whether there are any effects of low temperature in tropical or subtropical climates.

The investigation of effect modification is critical for identifying vulnerable subpopulations. A few studies have had inconsistent conclusions regarding the effect modification by age and sex [15,19–20]. For example, Chan et al. discovered females had a greater heat-related increase in AECOs than males in Hong Kong [16], whereas the converse sex difference was observed in Kaunas, Lithuania [20]. Some studies have found the elderly are more vulnerable to hot effects [19,21], but effect modification by age or sex was not found in other studies [15,22]. Additionally, the literature has indicated that the effect estimates vary by cities and climates. Additional studies on different regions and populations would improve the understanding of the potential impacts of temperature on emergency services.

Based on 120 AECOs data for the total population in the subtropical city of Shenzhen, China from 2010–2016, this study firstly aimed to examine the non-linear and lagged effects of high temperature and low temperature on AECOs, and secondly aimed to investigate the effect modification by age and sex.

## Materials and methods

### Study area and population

Shenzhen is one of the four first-tier cities in mainland China, situated approximately 1° south of the Tropic of Cancer, within the Pearl River Delta, and bordering Hong Kong to the south (22°33'N, 114°06'E). The municipality covers an area of 1,997.47 km^2^, including ten districts [23]. According to the 2016 Shenzhen Statistical Yearbook [24] and the Shenzhen 13th Five-Year Plan [25], the total population is 18.62 million, including 11.38 million permanent residents and approximately 7.24 million temporary residents, with a high population density of 9,321 per km^2^. Shenzhen has a GDP per capita of RMB ¥183,100 (USD $27,100) in 2017 [26] and ranks number one in mainland China.

Shenzhen has a subtropical monsoon climate with high humidity and extremely high temperatures during its long summer. On average, 135 days annually have a daily maximum temperature higher than 30°C. The long-duration summer significantly affects physical and mental comforts. The daily minimum and mean temperature are higher than 0°C year-round.

### Data collection

In mainland China, 120 is a specialized medical emergency telephone number. Shenzhen has a matured 24-hour 120 EMS system. This EMS is a unified and unique communication command system responsible for receiving all 120 emergency calls in the city and directing those calls to the closest hospital for ambulance dispatch. The system involves 83 hospitals, including state and private hospitals, with EDs and provide 120 ambulance emergency services in a radius of 3–5 km. A total of 150 ambulances transport patients, part of which resemble mobile intensive care units well equipped with monitoring instruments for resuscitation of critically ill patients [27–28].

Since 1997, all 120 AECOs in Shenzhen have been recorded in the EMS system. Notably, in mainland China, emergency ambulance services are provided for a fee, and the medical facilities and healthcare staff are relatively insufficient. These two factors limit the use of ambulance services.

We obtained all de-identified records in Shenzhen from 2010–2016 from the EMS. The records were summarized into daily counts of AECOs and stratified by sex and age groups. We considered four age groups (i.e., <15 years, 15–35 years, 35–65 years, and ≥65 years), similar to the literature [29]. Daily meteorological data during the same period, including daily ambient temperature (minimum, maximum and mean), mean sunshine duration and relative humidity were downloaded from the China Meteorological Data Sharing Service System [30].

### Statistical analysis

A quasi-Poisson regression model combined with distributed lag nonlinear model (DLNM) was applied to estimate the nonlinear and lag effects of ambient temperature on daily AECOs [31]. Some potential confounders were controlled, including sunshine duration, relative humidity, holiday, day of the week, seasonality and long-term trend of AECOs. The model was specified as follows:

$$\begin{array}{l} \log E[Y_{t}]=\alpha +\beta Temp_{t,l}+NS\left(Time_{t},7\times 8\right)+NS\left(SD_{t},3\right)\\ \;+NS(RH_{t},3)+\varepsilon Holiday_{t}+\gamma DOW_{t} \end{array}$$

where *t* is the day of the observation (t = 1,2,3…2 557), *Y*_*t*_ is the observed counts of daily AECOs, and *E*(*Y*_*t*_) is the expected number of AECOs on day *t*; α is the intercept; *Temp*_*t*,*l*_ is a cross-basis matrix of ambient temperature obtained by a “natural cubic spline-natural cubic spline.”

The natural cubic spline is constructed of piecewise third-order polynomial functions by dividing the domain of the independent variable into contiguous intervals and representing it by a separate polynomial in each interval. The number of knots (or degrees of freedom, df) is an important parameter to determine the number of intervals. DLNM with 5 *df* for temperature space (knots at equally spaced values in the range of temperature) and 4 *df* for lag space (knots at equally spaced log values) [32], and *l* is the maximum lag days; *NS*() is the natural cubic spline with 8 *df* per year for controlling the long-term trends and seasonality, and 3 *df* at equally spaced quantiles for the sunshine duration (*SD*) and relative humidity (*RH*) on the current day, as used in the literature [31]. For the categorical variables, *holiday* is the public holiday (0 for working days, 1 for Chinese Spring Festival, and 2 for other holidays), and *DOW* is the day of the week on day *t*; *β*, ε and *γ* are the coefficients.

Compared with other measures of daily temperature (e.g., maximum or minimum temperatures) and another specification of *df* for time, the model on daily mean temperature with a *df* of 8 for time generally showed the lowest Akaike’s Information Criterion for quasi-Poisson (S1 Table), which was therefore chosen for the subsequent analyses. Additionally, mean temperature indicates the exposure throughout the day, and the corresponding result is easier to interpret in a policy context. We set a maximum lag of 28 days in order to fully explore the lag structure of the temperature effect on AECOs. The cold and hot effects were defined as the relative risk (RR) of the AECOs for the 5th (12.8°C) and 95th (29.9°C) percentiles of temperature compared with 19.5°C, which was the relatively comfortable temperature with the minimum risk of AECOs. The extreme cold and hot effects were estimated by using the RR by comparing the 1st (8.9°C) and 99th (30.8°C) percentiles of temperature compared with 19.5°C. Furthermore, stratified analysis was performed by sex and age group.

Several sensitivity analyses were performed to examine the robustness of the main model. We tried alternative temperature measurements (i.e., daily maximum and minimum temperatures), changed *df* for time (range: 6–9), and used 4-day moving average (lag 0–3) instead of the current-day level of sunshine duration and relative humidity.

All statistical analyses were performed in the R 3.1.2. The “dlnm” package was used for fitting DLNM model. All statistical tests were two-sided, and *P*<0.05 was considered statistically significant.

## Results

**Fig 1:**
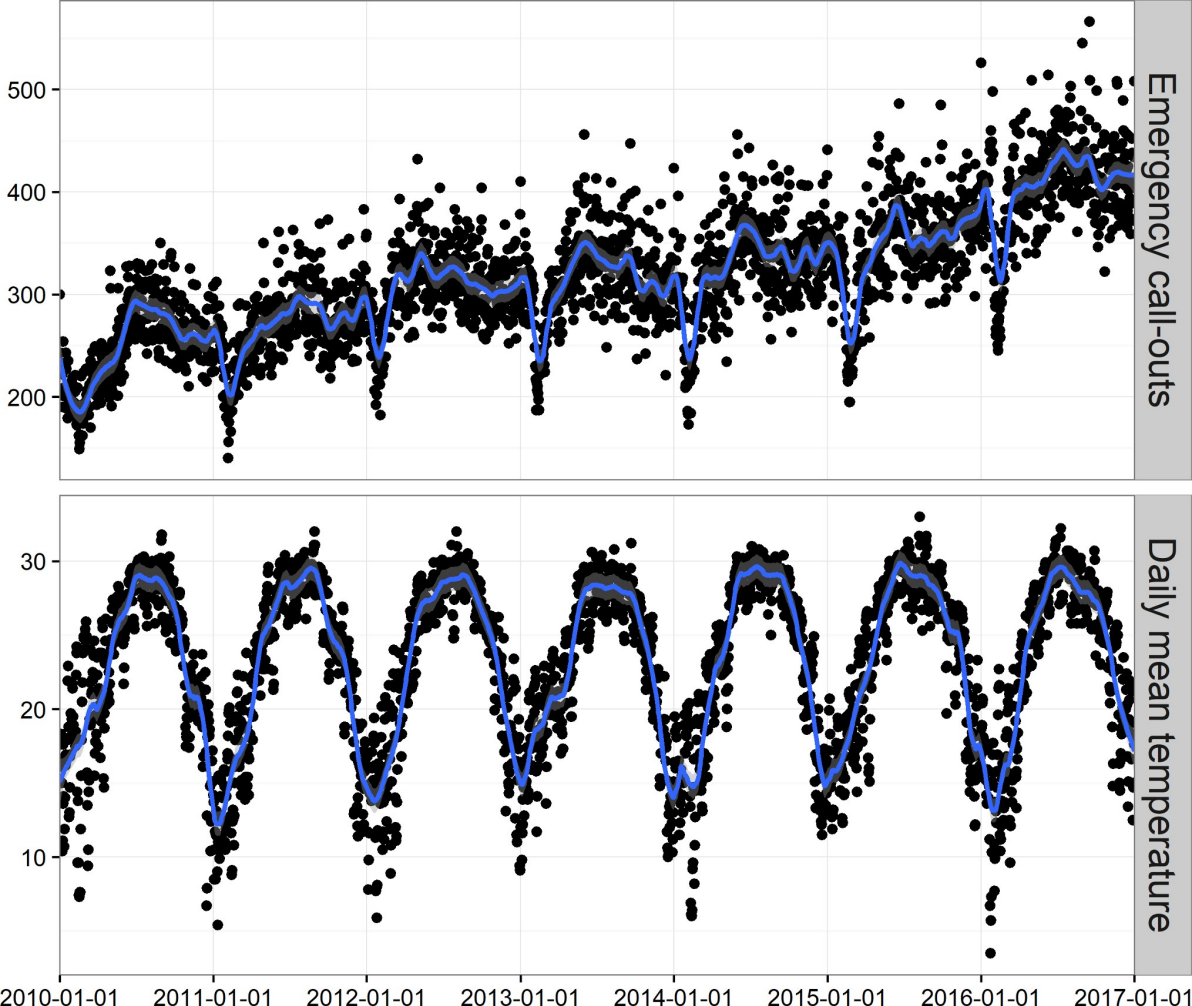
Time series of daily ambulance emergency call-outs and mean temperature during 2010–2016 in Shenzhen, China. The blue curve is the loess smoothing with a span of 3.5%.

Table 1 shows the descriptive statistics of daily AECOs and weather conditions during 2010–2016 in Shenzhen, China. There were 809,906 total AECOs during the study period with an average of 316.7 cases per day. The average daily AECOs were 14.8, 147.3, 115.2, and 33.1 cases for people aged <15, 15–35, 35–65 and ≥65 years, respectively. There were 199.1 male and 117.1 female cases per day. The averages of daily mean temperature, relative humidity, and sunshine duration were 23.2°C, 74.5%, and 5.2 hours, respectively. The time series of daily AECOs and mean temperature are shown in Fig 1. AECOs presented an increasing trend over the study period and an obvious seasonal pattern with peaks in summer and troughs in winter.

**Table 1:** Descriptive statistics of daily ambulance emergency call-outs and weather conditions from 2010–2016 in Shenzhen, China.

	Mean	SD	Min	25th	50th	75th	Max	Total
**Daily number of emergency call-outs**								
Age (years)								
<15	14.8	5.8	1	11	14	18	41	37 950
15–35	147.3	31.8	51	127	147	167	287	376 710
35–65	115.2	30	42	93	114	136	205	294 626
≥65	33.1	11.2	8	25	32	40	83	84 547
Sex								
Male	199.1	42.1	73	171	196	226	393	509 099
Female	117.1	24.6	54	100	116	133	215	299 408
Total	316.7	64	140	273	312	358	566	809 906
**Daily meteorological Measure**								
Maximum temperature (°C)	26.8	5.7	6.5	22.8	28.1	31.5	36.7	–
Mean temperature (°C)	23.2	5.7	3.5	19.0	24.7	28.0	33.0	–
Minimum temperature (°C)	20.8	5.7	1.7	16.6	22.2	25.6	29.8	–
Relative humidity (%)	74.5	12.9	19.0	68.0	76.0	83.0	100.0	–
Sunshine duration (h)	5.2	3.8	0.0	1.3	5.6	8.6	12.5	–

The SD represents standard deviation; the 25th, 50th and 75th represents the 25th, 50th, and 75th percentiles of distribution, respectively.

**Fig 2:**
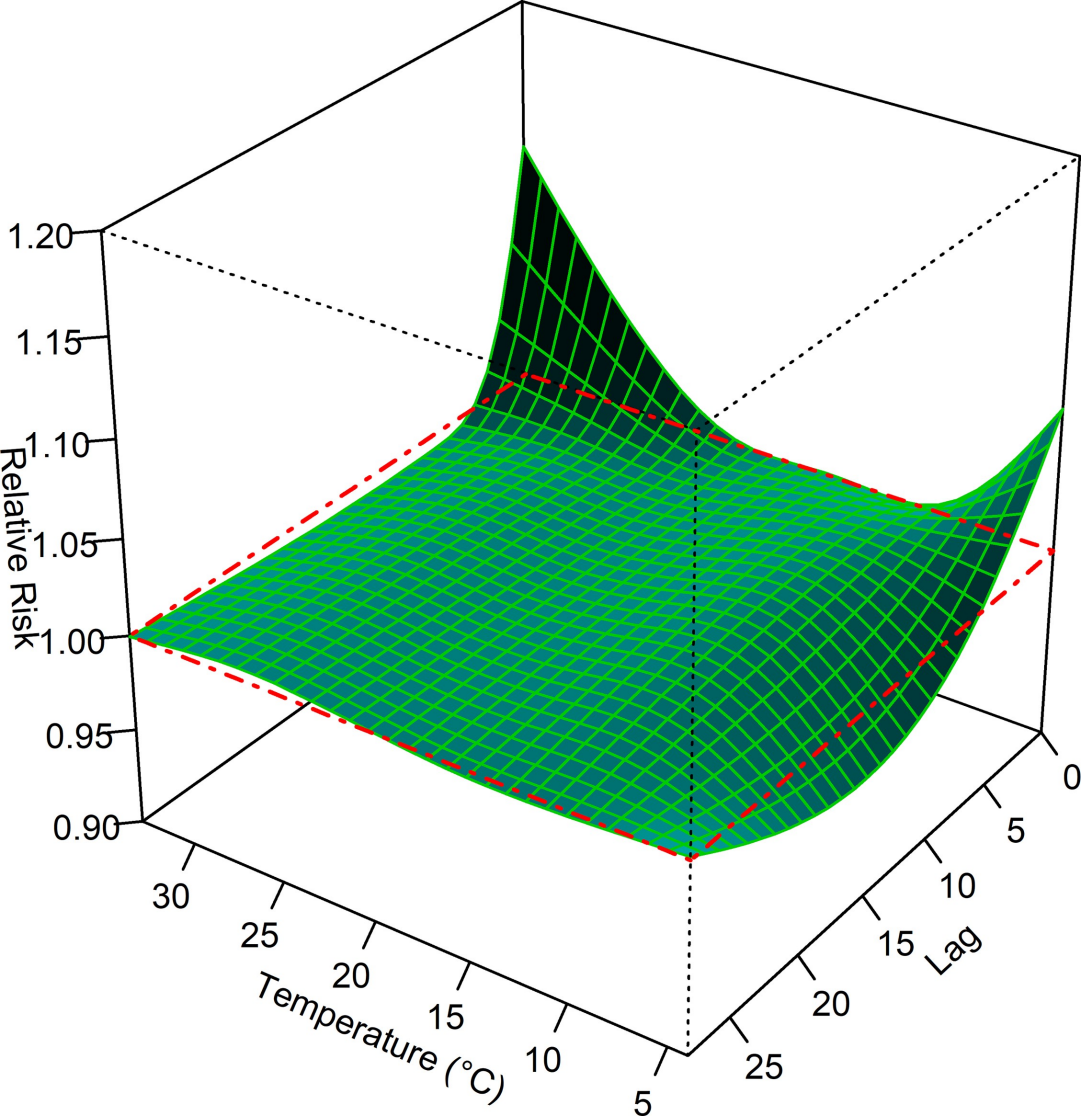
Relative risks of ambulance emergency call-outs along daily mean temperature and lag days. (A) The reference temperature is 19.5°C. (B) The cross section surrounded with dashed lines represents a relative risk (RR) of 1.

Fig 2 shows the 3-D plot for RR of AECOs along temperature and its lag days. A U-shaped relationship between daily mean temperature and daily counts of AECOs was observed, with increasing AECOs associated with low and high temperatures. The lag effects of temperature were observed, and lag patterns varied across temperature levels. A harvesting effect for extremely low, but not for high, temperatures was observed, with a protective effect from lag day 5.

**Fig 3:**
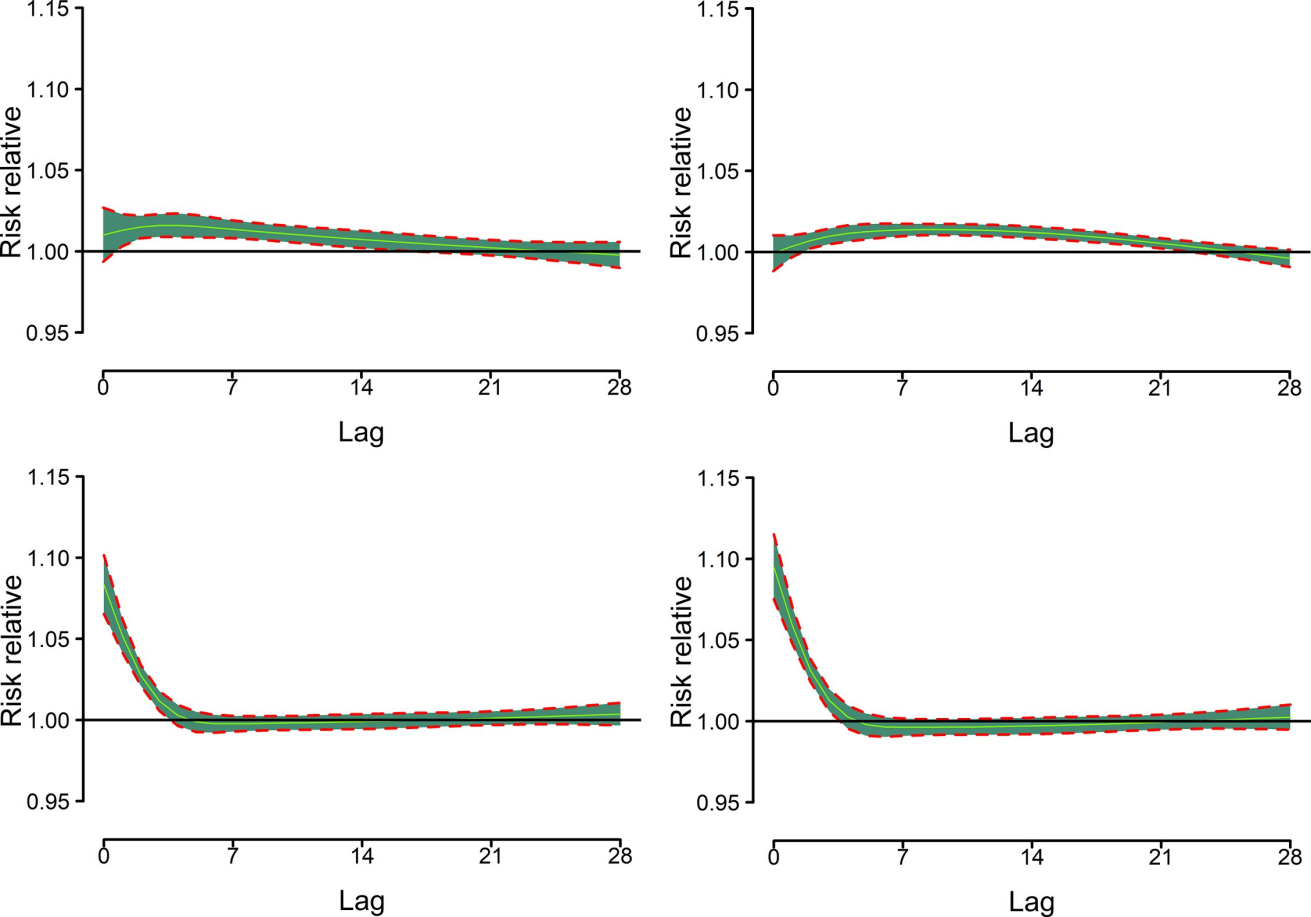
Relative risks (95% CI) of ambulance emergency call-outs along lag days. (A) Reference temperature is 19.5°C. (B) 8.9°C (top left), 12.8°C (top right), 29.9°C (bottom left), and 30.8°C (bottom right) are the 1st, 5th, 95th, and 99th percentiles of temperature distribution, respectively.

Fig 3 shows that cold effects lagged much longer than hot effects. Cold effects were the strongest at lag 5 and the effects persisted for approximately 3–4 weeks with a cumulative RR over lag 0–28 of 1.25 (95%CI: 1.16–1.35) and 1.23 (95%CI: 1.10–1.38) for cold effects and extreme cold impacts, respectively. The strongest hot effects were observed immediately on the current day (lag 0), and the effects almost disappeared after 5 days with a cumulative RR over lag 0–5 of 1.19 (95% CI: 1.14–1.23) and 1.21 (95% CI: 1.16–1.26) for hot effects and extreme hot effects, respectively. Significant cumulative effects of hot over lag 0–28 were also observed (Table 2).

**Table 2:** Cold and hot effects on daily ambulance emergency call-outs by age and sex from 2010–2016 in Shenzhen, China.

	Extreme cold effect*	Cold effect*	Hot effect*	Extreme hot effect*
**Lag 0–5 days**				
Total	1.09 (1.05–1.13)	1.04 (1.02–1.07)	1.19 (1.14–1.23)	1.21 (1.16–1.26)
Age group (years)				
<15	1.17 (1.03–1.34)	1.13 (1.03–1.23)	1.07 (0.95–1.21)	1.06 (0.93–1.21)
15–35	1.07 (1.01–1.13)	1.05 (1.01–1.09)	1.30 (1.23–1.37)	1.33 (1.26–1.41)
35–65	1.07 (1.02–1.13)	1.02 (0.99–1.06)	1.11 (1.06–1.17)	1.14 (1.08–1.19)
≥65	1.22 (1.14–1.32)	1.09 (1.03–1.15)	1.02 (0.95–1.11)	1.04 (0.96–1.14)
Sex				
Male	1.09 (1.04–1.14)	1.05 (1.02–1.09)	1.19 (1.14–1.24)	1.21 (1.15–1.27)
Female	1.10 (1.05–1.16)	1.03 (1.00–1.07)	1.18 (1.13–1.24)	1.21 (1.15–1.28)
**Lag 0–28 days**				
Total	1.23 (1.10–1.38)	1.25 (1.16–1.35)	1.19 (1.07–1.32)	1.17 (1.04–1.31)
Age (years)				
<15	1.49 (1.01–2.19)	1.56 (1.22–1.99)	1.09 (0.77–1.54)	1.02 (0.70–1.49)
15–35	1.20 (1.02–1.43)	1.27 (1.15–1.42)	1.26 (1.09–1.46)	1.26 (1.07–1.47)
35–65	1.22 (1.06–1.42)	1.24 (1.13–1.36)	1.14 (0.99–1.30)	1.14 (0.98–1.32)
≥65	1.44 (1.15–1.80)	1.24 (1.07–1.44)	1.22 (0.97–1.54)	1.18 (0.91–1.52)
Sex				
Male	1.24 (1.07–1.43)	1.29 (1.18–1.41)	1.18 (1.04–1.34)	1.15 (1.00–1.32)
Female	1.24 (1.07–1.43)	1.19 (1.08–1.30)	1.22 (1.07–1.39)	1.22 (1.06–1.41)

* The effects of extreme cold, cold, hot, and extreme hot were estimated using relative risks (95%CI) of ambulance emergency call-outs by comparing the risk of ambulance emergency call-outs for the 1st, 5th, 95th, and 99th percentile of temperature distribution, comparing with the reference temperature (19.5°C), respectively.

Table 2 presents effect estimates for subgroups by age and sex. Cold adversely affected all age groups with remarkable effects over lag 0–28. A particularly great cold effect was observed among children (aged <15), with a RR of 1.56 (95%CI: 1.22–1.99); notably, much higher than other age groups. The strongest effects of extreme low temperature was observed among children and the elderly (aged ≥65), with RRs of 1.49 (95%CI: 1.01–2.19) and 1.44 (95%CI: 1.15–1.80), respectively. Hot effects were significant and greater in the middle-aged groups, with a lag 0–5 RR of 1.30 (95% CI: 1.23–1.37) and 1.11 (95%CI: 1.06–1.17) for people aged 15–35 and 35–65 years, respectively. Hot effects were not statistically significant among children and the elderly. Compared with hot effects, extreme hot effects exhibited the same pattern but slightly greater differences among the age groups were observed. Cold effects and hot effects were significant for males and females, and sex differences in the effect estimates were very small.

Sensitivity analyses were performed to assess whether the results were robust to the specification of parameters in the model. We did not observe substantial differences in cold and hot effects by using daily minimum or maximum temperature instead of mean temperature, that is, changing df of time from 6 to 9, and controlling the potential confounding of relative humidity and sunshine duration by using the 4-day moving average (S1 and S2 Figs).

## Discussion

Shenzhen, China, has a matured EMS system and available EMS data. These two factors guarantee that our study, as a population-based investigation, provides a comprehensive analysis of the effect of ambient temperature on AECOs. This analysis contributes to an improved understanding of the nonfatal health impacts of ambient temperature. Based on AECOs from 2010–2016 in Shenzhen, we observed a nonlinear and lag effect of daily mean temperature on the frequency of AECOs, with substantially increasing AECOs associated with low and high temperatures. Cold effects delayed and persisted for 3–4 weeks. Hot effects were immediate and lasted for approximately 5 days. Children and the elderly were more vulnerable to cold effects. The youth and the middle-aged groups suffered more from the hot temperatures. The effects of cold and hot were similar for males and females.

We observed a U-shaped relationship between temperature and AECOs with an increase of 25% and 19% in the frequency of AECOs, comparing with the 5th and 95th percentile of temperature to the optimal temperature (i.e. 19.5°C, approximately the 27th percentile of the temperature distribution). Few studies have consistently reported an increase AECOs associated with high temperature [14–15,22,33–35]. The optimal temperature and the magnitude of effect estimates were different. The difference was probably because of differences in time scale, lag used, and population acclimation to various climates. For example, Guo et al. [15] found a U-shaped relationship for temperature and AECOs in Brisbane, Australia, on an hourly time scale and reported a lag 0–48 hours increase of 29% for the 99th percentile of temperature (33°C), compared with the 85th percentile of temperature (28.7°C). Additionally, Jegasothy et al. [14] found a heat wave increased 10.9% of AECOs within 7 days in New South Wales.

Although the literature has reported significant cold effects on mortality in subtropical cities [36–37], only one study reported cold effects on AECOs in a subtropical climate: a 1°C decrease below 15.5°C caused a 1.30% (0.87% to 1.73%) increase in total AECOs in Brisbane, Australia [38]. In addition, low temperature was shown to significantly increase hospital ED visits in some subtropical cities [10,12,39], and cold effects on ED visits were not observed in Shanghai, China [33].

Understanding the lag characteristic of temperature effect on AECOs is important to improve the efficiency of emergency ambulance service planning. The literature on ambulance dispatches has generally pre-specified a maximum lag and reported the cumulative effect of temperature without clear data of lag patterns [16,40].

In this study, we examined the lag patterns and observed high-temperature elevated AECOs on the current day and following 5 days, whereas low temperature increased AECOs gradually and persisted for approximately 3–4 weeks. Another study in Guangzhou found that the significant hot effects on ambulance emergency dispatches for renal colic appeared acutely and lasted for 5 days [18]. Many examples in the literature on ambient temperature and the morbidity/mortality relationship have also shown that hot effects were immediate and diminished quickly (several days), and cold effects were observed later and persisted much longer (up to 3–4 weeks) [2,5–6,41].

Identification of susceptible subpopulations would help develop targeted measures to protect these subgroups from the adverse impacts of extreme weather. We found children and the elderly were more vulnerable to low temperature, and this result is consistent with the results from numerous studies on the temperature–mortality association [2,42–43]. In the south of China, air conditioning is commonly used, but home heating equipment is rarely used; thus, the population is commonly exposed to low temperatures in winter. The weaker physique of children and the elderly and a higher prevalence of comorbidities in the elderly put them at a higher risk of suffering from cold weather [2,43].

We also found that 120 call frequency among the youth and middle-aged was more sensitive to high temperature, and this result is similar with the findings on AECOs in King County, Washington (US) and Adelaide, Australia [44–45] and on heat-related ED visits in Rhode Island (US) [46]. Compared with children and the elderly, the youth and middle-aged live a faster-paced lifestyle and suffer greater stresses from living and working, that is, they must go out for business, sometimes work outdoors (e.g., construction workers), and do more strenuous exercises outside, all of which increase their exposure to high temperature and result in a higher risk of emergency medical events during hot days, although they are generally considered to be relatively resilient. The youth and middle-aged are also the main labor force and principal users of the EMS system, accounting for 82.9% of all AECOs in our study; therefore, specialized publicity and measures should be taken to protect them from the harm related to high temperatures.

The temperature–AECOs association was found to be similar between males and females in this study; however, the literature was not consistent regarding the effect modification of the temperature–AECOs association by sex [16,20] across cities. This phenomenon might be caused by the features of different cities and populations (e.g., climate, geographic features, education level, economic position, infrastructure construction, and health policy).

Despite the absence of a clear mechanism of the impacts of ambient temperature on AECOs, there are several possible explanations. Extremely low and high temperatures stress the thermoregulatory system and increase blood pressure, platelet and red cell counts, blood viscosity, and blood cholesterol levels, increasing the risk of arterial thrombosis [47–48]. The main cause of AECOs is road traffic trauma, accounting for 46.6% of AECOs in 2011 in Shenzhen, China [28]. Both extreme cold and hot can affect traffic volume, and driving performance (e.g. drivers’ physiological responses and speed variability) [49–50], and the friction of roads because ambient temperature changes tire pressure, rubber flexibility, and road conditions [51–52], increasing the risk of traffic trauma. The findings regarding the temperature–AECOs association have important implications for public health education: individuals can adapt their practices to improve health and resilience against the negative impacts of cold or hot weather. The full understanding of the characteristics of the association provide important information for further establishing a prediction and warning system of medical emergency needs based on weather forecasts. A satisfactory EMS system should be sensitive to extreme temperatures and prepare a sufficient number of ambulances and medical staff in advance for the sudden increasing in AECOs.

This study has some limitations. First, this was a single-city study; thus, caution should be used before generalizing the effect estimates to other regions, particularly to rural areas, because the health service needs and utilization may differ from the urban areas. Second, we failed to obtain diagnosis information for the cause-specific analysis of ambulance call-outs, because the 120 EMS system is independent of hospital systems and includes only preliminary and unreliable diagnosis information for patients. Third, after adjusting for meteorological confounders, we cannot exclude the possibility of other residual confounding. The levels of air pollution were not controlled in the model because of the data were unavailable. Notably, the literature on the temperature–mortality associations has shown that the confounding effect of air pollution was nonsignificant [53–54].

In the future, multi-city studies could provide evidence that is more conclusive, regarding the temperature–AECOs association. Further efforts could be taken to link the 120 data to the medical records of patients after being transported to a hospital. Combining prehospital data, and accurate diagnosis information, and detailed socio-economic data would help identify vulnerable populations.

## Supporting information

S1 Table Quasi-likelihood Akaike information criteria for the effect of ambient temperature on AECOs by temperature measure and degree freedom of time. (DOCX)

S1 Fig The 3-D plot of the association between ambient temperature and AECOs. The left one is Maximum temperature, and the right one is minimum temperature.
(TIFF)

S2 Fig The 3-D plot of the association between daily mean temperature and AECOs. The top-left, top-right and bottom-left are degrees of freedom of 6, 7 and 9 for time, respectively. The bottom-right controlled for 4-day moving average of relative humidity and sunshine duration.
(TIFF)

S1 File Full raw dataset of the study. (CSV)

S2 File Analytical program of core model in R language. (R)
