# Supplementary material for: Effects of ambient temperature on ambulance emergency call-outs in the subtropical city of Shenzhen, China
Source: PLoS One. 2018 Nov 12;13(11):e0207187. doi: 10.1371/journal.pone.0207187 (PMC6231653; doi:10.1371/journal.pone.0207187)
Supplement: S1 Table — (DOCX) [file pone.0207187.s001.docx]

**S1 Table.** **Quasi-likelihood Akaike information criteria for the effect of ambient temperature on AECOs by temperature measure and degree freedom of time.**

| **Temperature measure** | **Degree freedom of time** | | | |
| --- | --- | --- | --- | --- |
|  | **5** | **6** | **7** | **8** |
| **Maximum temperature** | 25514 | 25408 | 25291 | 25204 |
| **Mean temperature** | 25521 | 25427 | 25281 | 25158 |
| **Minimum temperature** | 25592 | 25497 | 25309 | 25174 |
