# Supplementary figures and images for: Effects of ambient temperature on ambulance emergency call-outs in the subtropical city of Shenzhen, China
Source: PLoS One. 2018 Nov 12;13(11):e0207187. doi: 10.1371/journal.pone.0207187 (PMC6231653; doi:10.1371/journal.pone.0207187)

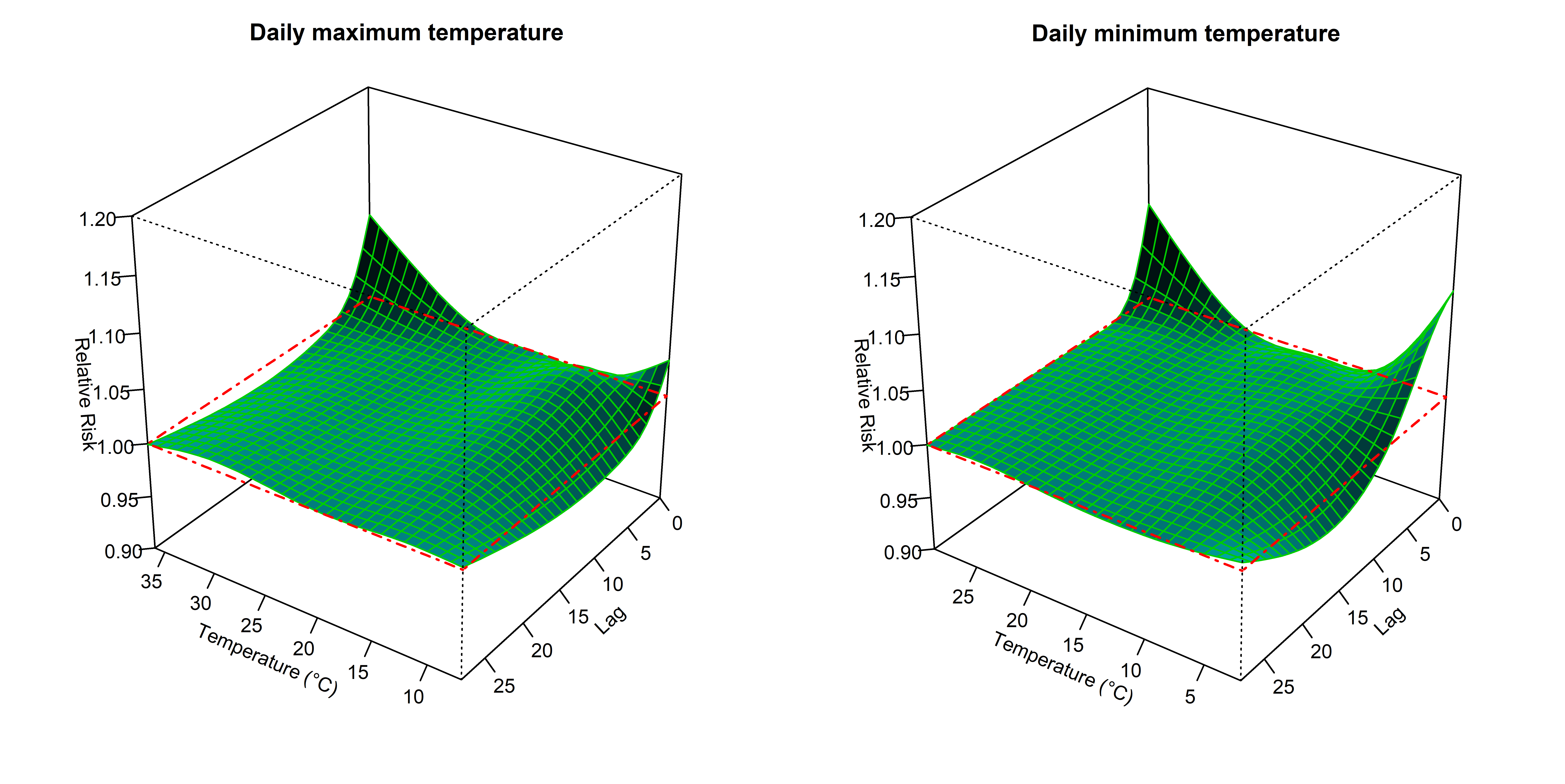

Supplement: S1 Fig — The left one is Maximum temperature, and the right one is minimum temperature. (TIFF) [file pone.0207187.s002.tiff]
